# Recruitment strategies and HPV self-collection return rates for under-screened women for cervical cancer prevention

**DOI:** 10.1371/journal.pone.0280638

**Published:** 2023-03-23

**Authors:** Jennifer S. Smith, Olivia M. Vaz, Charley E. Gaber, Andrea C. Des Marais, Bhavika Chirumamilla, Lori Hendrickson, Lynn Barclay, Alice R. Richman, Xian Brooks, Anna Pfaff, Noel T. Brewer

**Affiliations:** 1 Department of Epidemiology, Gillings School of Global Public Health, University of North Carolina at Chapel Hill, NC, United States of America; 2 Lineberger Comprehensive Cancer Center, University of North Carolina, Chapel Hill, NC, United States of America; 3 Hospital Epidemiology, University of North Carolina Medical Center, Chapel Hill, NC, United States of America; 4 American Sexual Health Association, Research Triangle Park, NC, United States of America; 5 Department of Health Education and Promotion, College of Health and Human Performance, East Carolina University, Greenville, NC, United States of America; 6 School of Nursing, University of Louisville, Louisville, KY, United States of America; 7 Department of Maternal and Child Health, Gillings School of Global Public Health, University of North Carolina at Chapel Hill, NC, United States of America; 8 Department of Health Behavior, Gillings School of Global Public Health, University of North Carolina, Chapel Hill, NC, United States of America; Kaiser Permanente Washington, UNITED STATES

## Abstract

In the United States, medically underserved women carry a heavier burden of cancer incidence and mortality, yet are largely underrepresented in cancer prevention studies. My Body, My Test is a n observational cohort, multi-phase cervical cancer prevention study in North Carolina that recruited low-income women, aged 30–65 years and who had not undergone Pap testing in ≥ 4 years. Participants were offered home-based self-collection of cervico-vaginal samples for primary HPV testing. Here, we aimed to describe the recruitment strategies utilized by study staff, and the resulting recruitment and self-collection kit return rates for each specific recruitment strategy. Participants were recruited through different approaches: either direct (active, staff-effort intensive) or indirect (passive on the part of study staff). Of a total of 1,475 individuals screened for eligibility, 695 were eligible (47.1%) and 487 (70% of eligible) participants returned their self-collection kit. Small media recruitment resulted in the highest number of individuals found to be study eligible, with a relatively high self-collection kit return of 70%. In-clinic in-reach resulted in a lower number of study-eligible women, yet had the highest kit return rate (90%) among those sent kits. In contrast, 211 recruitment which resulted in the lowest kit return of 54%. Small media, word of mouth, and face-to-face outreach resulted in self-collection kit return rates ranging from 72 to 79%. The recruitment strategies undertaken by study staff support the continued study of reaching under-screened populations into cervical cancer prevention studies.

## Introduction

In the United States, overall cancer mortality rates have steadily declined since the 1990s [1]. However, racial and ethnic minorities and medically underserved women- those of low socioeconomic status (SES), racial or ethnic minorities, or non-English speaking- continue to carry a heavier burden of cancer incidence, morbidity, and mortality [1–3]. Blacks are 14% more likely to die of any cancer than whites [1]. Over half of new invasive cervical cancer cases occur in under-screened women [1, 2, 4]. Invasive cervical cancer has notable health disparities, with low-income and ethnic minority women having higher mortality rates [5].

To develop appropriate interventions for cervical cancer control, it is critical that recruitment and participation in cancer research ensure the proper representation of racial and ethnic minorities [6]. In a 2012 study of minority participation in cervical cancer studies, enrollment of Black patients was 4.5-fold lower than anticipated [7]. Potential barriers to research participation also include income and insurance status [8–10]. Low-income individuals are over 25% less likely to participate in clinical cancer trials, and insured individuals are 4 times more likely to participate than their uninsured counterparts [8, 11]. Efforts to increase participation in cancer prevention research among higher risk populations are needed at all levels: at the primary prevention level to reduce the incidence of HPV-associated precancers by vaccination, at the secondary level to increase screening for early detection before progression to cancer, and at the tertiary level to improve treatment to reduce mortality rates. There is an identified need for effective methods to recruit ethnic minority women to research studies [12].

Researchers have been increasingly quantitatively documenting their experiences with the recruitment and retention of medically underserved populations into clinical research and cancer prevention studies [13–15]. However, existing literature on strategies to improve underserved population representation in biomedical research is mainly descriptive, and few strategies have been rigorously tested [14–16]. To better describe actions taken in recruitment, the comparison of “indirect” versus “direct” recruitment strategies has been utilized. These “direct” versus “indirect” methods are grounded in the previously defined concept of active versus passive recruitment methods [12]. Active (“direct”) approaches are characterized by researchers targeting specific individuals, groups, or residents and recruiting from a subject pool. In contrast, passive (“indirect”) recruitment, the researcher makes the target population aware of the study, but leaves it up to potential participants to approach the researcher to participate [12].

Here, we aimed to describe the recruitment strategies utilized by study staff, and the resulting recruitment and self-collection kit return rates for each specific recruitment strategy. Results are presented on the methods used in recruiting medically underserved women into the My Body, My Test cervical cancer prevention studies. Two phases of the My Body, My Test study (MBMT-1 and -2) investigated a home-based, self-collection screening approach to test for high-risk (oncogenic) human papillomavirus (HPV) infection—the virus that is the primary cause of cervical cancer. We describe here the recruitment strategies undertaken to reach recruit a diverse population of medically-underserved individuals, as well as the recruitment numbers and self-collection kit return rates yielded with each recruitment strategy.

## Materials and methods

### Study overview and sample

My Body My Test is an observational cohort, multi-phase study investigating the use of home-based self-collection of samples for high-risk HPV testing among women who are under-screened for cervical cancer. Phase one (MBMT-1) assessed the feasibility of home-based self-collection in a low-income, under-screened population as previously reported [17, 18]. Phase two (MBMT-2) assessed self-collection test validity by comparing HPV test results from home-based self-collected samples to in-clinic clinician-collected samples [19]. Here, we report on the recruitment strategies utilized across the two study phases.

In brief, MBMT1 participants were recruited from 10 North Carolina counties identified as having relatively high rates of cervical cancer: Wake, Durham, Harnett, Guilford, Wayne, Cumberland, Robeson, Richmond, Hoke, and Scotland counties [20]. Recruitment occurred from January 2010 to September 2011. Women were eligible if they were aged 30–65 years, not pregnant, without Pap test in the preceding 4 or more years, without history of hysterectomy, and low-income [income criteria: children qualifying for the federal school lunch program, Medicaid or Medicare Part B insurance, or uninsured and living at or below 200% of the federal poverty level (determined by household income and size)].

From February 2012 to October 2014, MBMT-2 participants were recruited from five North Carolina counties: Alamance, Buncombe, Chatham, Durham, and Orange. Female residents of these counties were eligible if they were aged 30–64 years, not pregnant, without Pap test in the preceding 4 years, without history of hysterectomy, earning below 250% of the poverty level (determined by household income and size), and uninsured, underinsured, or insured through Medicaid.

If a woman was found eligible for MBMT-1 and -2, she was mailed an at-home self-collection kit containing the Viba self-collection brush (Rovers), illustrated instructions, and a stamped return envelope [17]. When the kit was returned, the self-collected cervico-vaginal sample was sent for laboratory testing, and participants were contacted to receive their HPV results. Kits were considered not returned if they were not received back for HPV testing within 18 months of receiving the kit.

This study was approved by the University of North Carolina at Chapel Hill Institutional Review Board (IRB). All participants provided written informed consent before study enrollment.

### Recruitment strategies

MBMT-1 and MBMT-2 utilized both direct and indirect participant recruitment strategies (See Details in Table 1). Direct strategies involved higher staff involvement for the active dissemination of information about the study. Face-to-face outreach included study staff visiting public venues to recruit potential participants. Another direct strategy was the use of a 211 social assistance hotline run by United Way of North Carolina. UNC study staff prepared a short script and trained 211 staff on the outreach screening study. 211 then offered information about the study to callers who met initial study eligibility criteria, as well as the number of the MBMT study hotline for women interested in the HPV study to call in order to undergo a full eligibility screening. MBMT-2 staff also actively recruited through clinic “in-reach,” or recruitment of new female patients identified as overdue for cervical cancer screening during in-person intake at a Federally Qualified Health Center (FQHC) (See Table 1). Direct recruitment using face-to-face outreach which specifically targeted Latina women was initiated during MBMT-2 after observing that this population was not sufficiently represented in the MBMT-1 study.

**Table 1:** Definitions of recruitment strategies.

Small Media	Indirect recruitment strategy: Small media included postcards, brochures, and posters developed for low-literacy comprehension that were distributed via mail, email, or in-person drop-off. Materials were provided to interested community partners such as churches, food banks, shelters, the Department of Social Services, clinics, supermarkets, and public housing units to distribute to their clients. Advertisements were also placed in local newspapers and the websites of collaborating organizations, and the study website provided additional information.
Word of Mouth	Indirect recruitment strategy: All eligible and ineligible women who contacted the study hotline were encouraged to inform potentially eligible family and friends about the study in order to maximize snowball sampling. Recruitment materials were also included in the mailed self-test kits, so that women could easily share this information.
211 referral	Direct recruitment strategy: Callers to the 2-1-1 social assistance hotline run by United Way of North Carolina who met initial eligibility criteria were offered information about the study and, if interested, were transferred to the study hotline for full eligibility screening and enrollment. This collaboration facilitated focused outreach to low-income women seeking social assistance. 2-1-1 is an information and referral helpline that is a federally designated dialing code that provides callers with referrals to local health and social service agencies. Callers to 2-1-1 speak with trained specialists that identify the caller’s needs, find local resources to provide assistance, and give information to help callers contact service providers for assistance [21].
Face-to-Face Outreach	Direct recruitment strategy: Staff visited public venues such as stores, restaurants, clinics, and laundromats to directly recruit potential participants. Face-to-face recruitment was also conducted through booths at community festivals, flea markets, and health fairs.
Clinic In-Reach (MBMT-2 only)	Direct recruitment strategy: Potentially eligible new and established patients overdue for cervical cancer screening were provided with information about the study when they came in for an office visit at Western NC Community Health Services (WNCCHS), a Federally Qualified Health Center (FQHC) in Buncombe County.

In contrast to direct strategies, indirect strategies were strategies that did not require intensive involvement by study staff and provided passive dissemination of information about the study. The most common indirect strategy was small media, in which UNC study staff called and mailed, or directly provided small media materials to interested community partners, or purchased local ads in newspapers or websites of collaborating organizations. Word of mouth recruitment was initiated by encouraging eligible and ineligible women who had contacted the study hotline to share information about the study with their friends and family.

Potential participants were screened for eligibility and enrolled using the study hotline, which was administered by call center staff from the American Sexual Health Association (ASHA). Most participating women were recruited directly by completing study eligibility screening with a hotline staff member. Women enrolled through clinic in-reach had their full information collected in person, and thus did not need to call the hotline to finish enrolling.

### Data collection

At the beginning of eligibility screening, all callers to the study hotline were asked, “how did you first hear about this study?” When participants were recruited directly, study personnel recorded this recruitment source. In the MBMT-1 study, variables of age and time since last Pap were collected to determine study eligibility during initial contact with the participant, and insurance status, race, educational level, and ethnicity were collected during a second call with participants after they were deemed eligible and sent a self-collected sample kit. In MBMT-2, demographic variables were collected during the initial eligibility screening on the phone with ASHA call center after they were deemed study-eligible. All eligibility data were entered and stored in a centralized, secure study database.

### Data analysis

We calculated the demographics of the population recruited, including categories for age, education, health insurance status, race, and ethnicity. Participant self-collection return rates were calculated, comparing kit return rates across recruitment sources, comparing the recruitment methods individually and grouped as indirect versus direct approaches, using a Pearson chi-square test. Statistical tests were considered significant at p = <0.05. Analyses were conducted using SAS 9.4 (SAS Institute Inc., Cary, NC).

## Results

**Fig 1:**
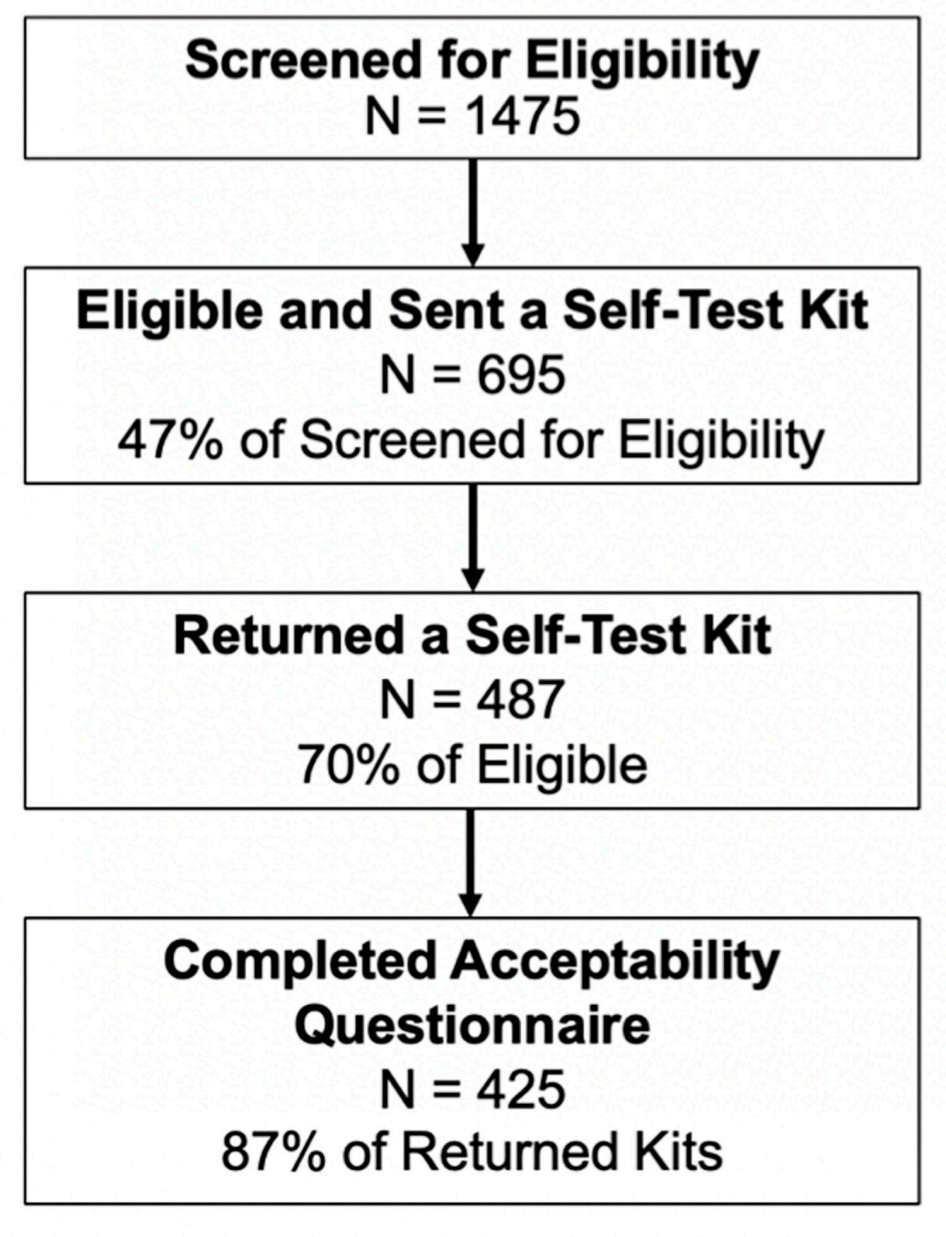
Overall progression of individuals through study.

Recruitment efforts resulted in a total of 1,475 individuals screened for study eligibility, 47.1% of whom were deemed eligible (n = 695) (Fig 1). All eligible women were mailed a self-collection kit, and overall, 70% of those eligible returned the kit (n = 487). Of those who returned their self-collection kit, 87% completed one of the study questionnaires (n = 425).

Of those recruited and eligible, approximately half (54.4%) were under 45 years of age (Table 2). Most eligible women were uninsured (74.6%) and had no higher than a high school education (61%). Over a third (39.3%) of those eligible were black, 40.4% were white, and 20.3% were classified as “Other” race, which included Asian, Native Hawaiian or Pacific Islander, American Indian or Alaska Native, or Hispanic. Roughly one-fifth (18.4%) were Latina (Table 2). Over half (62.3%) of individuals aged <45 years were recruited via indirect strategies. No Latina participants were recruited via clinic in-reach, nor were any individuals classified as “Other” racially.

**Table 2:** Proportions of study-eligible participants’ recruited into the in my body my test phases 1 and 2 study, stratified by demographics of eligible participants.

			Indirect Recruitment Strategies		Direct Recruitment Strategies		
Recruitment Method			%, n		%, n		
			95% confidence intervals		95% confidence intervals		
			Small Media†	Word of Mouth†	211 Referral†	Face-to-Face Outreach†	Clinic In-Reach†
		**Total (n = 695)**	41.4%, n = 288	17.3%, n = 120	22.6%, n = 157	11.5%, n = 78	7.2%, n = 50
			(38–45%)	(15–20%)	(20–26%)	(9–14%)	(5–9%)
**Age in years**	**<45**	377 (54.4%)*	40.8%, n = 154	21.5%, n = 81	22.5%, n = 85	10.9%, n = 41	4.2%, n = 16
			(36–46%)	(17–26%)	(18–27%)	(7.9–14%)	(2.5–6.8%)
	**≥45**	316 (45.6%)*	42.1%, n = 133	12.3%, n = 39	22.5%, n = 71	12.3%, n = 39	10.8%, n = 34
			(37–48%)	(9–16%)	(18–28%)	(8.9–16%)	(7.6–15%)
**Insurance Status**	**Insured**	107 (25.4%)*	38.3%, n = 41	17.8%, n = 19	27.1%, n = 29	3.7%, n = 4	13.1%, n = 14
			(29–48%)	(11–26%)	(19–37%)	(1.0–9.3%)	(7.3–21%)
	**Uninsured**	314 (74.6%)*	42.4%, n = 133	18.5%, n = 58	13.4%, n = 42	16.2%, n = 51	9.6%, n = 30
			(37–48%)	(14–23%)	(9.8–18%)	(12–21%)	(6.5–13%)
**Education**	**≤ High school**	291 (61.0%)*	39.5%, n = 115	19.6%, n = 57	14.8%, n = 43	17.5%, n = 51	8.6%, n = 25
			(34–45%)	(15–25%)	(11–19%)	(13–22%)	(5.6–12%)
	**> High School**	186 (39.0%)*	50.5%, n = 94	10.2%, n = 19	19.9%, n = 37	5.9%, n = 11	13.4%, n = 25
			(43–58%)	(6.3–15%)	(14–26%)	(3.0–10%)	(8.9–19%)
**Race**	**White**	223	44.8%, n = 100	13.0%, n = 29	19.3%, n = 43	4.5%, n = 10	18.4%, n = 41
		(41.5%)*	(38–52%)	(8.9–18%)	(14–25%)	(2.2–8.1%)	(14–24%)
	**Black**	217 (39.3%)*	50.7%, n = 110	19.8%, n = 43	19.8%, n = 43	5.5%, n = 16	4.1%, n = 5
			(44–58%)	(15–26%)	(15–26%)	(4.3–12%)	(0.8–5.3%)
	**Other**	112 (19.9%)*	23.2%, n = 26	17.9%, n = 20	7.1%, n = 8	51.8%, n = 58	0.0%, n = 0
			(16–32%)	(11–26%)	(3.1–14%)	(42–61%)	(0–3.2%)
**Ethnicity**	**Latina**	94 (18.4%)*	12.8%, n = 12	20.2%, n = 19	5.3%, n = 5	61.7%, n = 58	0.0%, n = 0
			(6.8–21%)	(13–30%)	(1.8–12%)	(51–72%)	(0–3.9%)
	**Non-Latina**	416 (81.6%)*	49.0%, n = 204	15.9%, n = 66	18.3%, n = 76	4.8%, n = 20	12.0%, n = 50
			(44–54%)	(12–20%)	(15–22%)	(3.0–7.3%)	(9–16%)

*Column percentages are presented; †Row percentages are presented. Counts may not sum to 695 (number of individuals screened and determined eligible) due to missing values or because participants did not reach the stage in the study where they were asked this question. Missing: Race: 143, Ethnicity: 185 Age: 2, Insurance: 274, Education: 218

### Recruitment methods: Proportion and return rates

Of the specific recruitment methods used, small media accounted for 41.1% of individuals screened and deemed study-eligible (Table 3), followed by 211 referral (22.6% of participants) and word of mouth (17.3%). Face-to-face outreach and clinic in-reach (the latter of which was only utilized in Phase 2 of recruitment in MBMT-2), each accounted for 11.5% and 7.2% of study-eligible participants, respectively. Clinic in-reach resulted in 7.2% of recruited patients and had the highest percentage of eligible individuals who returned their kit (90%), the highest kit return rate of all recruitment strategies. Small media, word of mouth, and face-to-face outreach each had self-collection kit-return rates above 70%: 71%, 72% and 79%, respectively. 211 referral was the only recruitment strategy to have a less than 70% self-collection return rate, with 54.1% of eligible patients returning their completed self-collection kit.

**Table 3:** Proportion and continued participation of participants, stratified by recruitment source, and indirect versus direct recruitment strategy.

			Indirect Strategies		Direct Strategies		
		Total	Small Media	Word of Mouth	211 Referral	Face-to-Face Outreach	Clinic In-reach
**Eligible and sent kit**	N	695	288	120	157	80	50
	Percent of all participants		41.4%	17.3%	22.6%	11.5%	7.2%
			(38–45%)	(15–20%)	(20–26%)	(9–14%)	(5–9%)
**Returned self-test kit**	N returned kit	487	208	86	85	63	45
	Percent of all participants who returned kits	--	42.7%	17.7%	17.5%	12.9%	9.2%
			(38–47%)	(14–21%)	(14–21%)	(10–16%)	(7–12%)
	Percent of eligible	70.1%	72.2%	71.7%	54.1%	78.8%	90.0%
			(66–77%)	(63–80%)	(46–62%)	(68–87%)	(78–97%)
**Completed questionnaire**	N completed survey	425	179	78	70	55	43
	Percent of all participants	--	42.1%	18.4%	16.5%	12.9%	10.1%
			(37–47%)	(15–22%)	(13–20%)	(10–17%)	(7–13%)
	Percent of eligible	61.2%	62.2%	65.0%	44.6%	68.8%	86.0%
			(56–68%)	(56–73%)	(37–53%)	(57–79%)	(73–94%)

† Clinic in-reach used only in the 2^nd^ study phase

MBMT-2. 95% Confidence intervals for each estimate are presented in brackets under a given percentage.

## Discussion

MBMT-1 and -2 resulted in relatively high self-collection kit return rates, with 70% of eligible participants returning their kits overall. Small media recruitment resulted in the highest number of individuals found to be study eligible, with a relatively high self-collection kit return of 70%. In-clinic in-reach resulted in a lower number of study-eligible women, yet had the highest kit return rate (90%) among those sent kits. In contrast, 211 recruitment which resulted in the lowest kit return of 54%. Small media, word of mouth, and face-to-face outreach resulted in self-collection kit return rates ranging from 72 to 79%.

Of the 695 under-screened women who were mailed HPV self-collection kits for cervical cancer screening, kit return was relatively high when compared to similar studies involving mailed HPV self-collection kits which resulted in lower kit return rates (15%-38%) [22–24]. The relatively higher observed self-collection kit return rates may be because our study relied more heavily on participants taking the initiative to contact the study after receiving recruitment information.

It is important to note that most chosen recruitment methods resulted in more than 70% of those recruited returning their self-collection kits. Small media recruitment resulted in the highest number of individuals found to be study eligible, with a relatively high self-collection kit return of 70%. Clinic in-reach resulted in the highest rate of patients returning their self-collection kit at 90% of eligible patients recruited via this method. This may be because these individuals were recruited while seeking healthcare services at the partner FQHC, and thus may have been more likely to complete cervical cancer screening. On the other hand, 211 referral had the lowest kit return rate, with just over half of eligible participants recruited via this method returning their self-collection kit. Because 211 referral was a collaboration with a social assistance hotline aiding low-income women, individuals may not have been able to complete the study due to other competing obstacles. Future studies should consider the balance of recruitment success by both self-collection kit return rates of participants and the representation of underserved populations in their surveyed study populations.

Difficulties in the recruitment of individuals who do not have regular or easy access to healthcare, or come from underserved populations has been well documented [8, 10, 11]. Barriers to recruitment include a lack of health insurance, language barriers, cultural beliefs, competing time demands, lack of resources (i.e. telephone, income), and logistical concerns (i.e. transportation, geographic distance) [13, 16, 25, 26]. Our study recruitment methods aimed to overcome these obstacles by utilizing retention facilitators, including timely incentives, phone reminders, and accessible study clinic locations to facilitate follow-up [14]. The MBMT studies were conducted to provide an accessible screening option to our target population of women overdue for screening, as self-collection kits were mailed to participants’ homes in order to facilitate convenience for participants. A large proportion of women recruited to participate in this study did not have health insurance (74.6%), representing women who would be aided by future programs using innovations such as self-collection kits to increase screening rates in underserved populations.

To our knowledge, there are limited data on the recruitment and participation of individuals in research on cervical cancer screening for underserved populations. A 1999 study aimed at describing recruitment strategies for a single-visit cervical cancer prevention study in a low-income, predominantly Latina population in California found that recruitment was higher and loss-to-follow-up lower in women recruited via media recruitment versus from a clinic registry [27]. Our results here also similarly showed that small media recruitment strategies, which included the distribution of postcards, brochures, and posters developed for low-literacy comprehension via mail, email, or in-person drop-off, appeared quite successful for recruitment of medically underserved women in our MBM studies. Small media advertising for our study was also relatively inexpensive, but did require some study staff time to distribute study-associated materials.

Recruitment and retention challenges were identified in a 2011 small, randomized bio-behavioral clinical trial of 50 cervical cancer survivors in California, although study participants needed have regular access to a phone to be eligible for the study [28]. In our study, we evaluated five different recruitment strategy methods. After observing that the Latina population was not sufficiently represented in the MBMT-1 study, targeted direct recruitment via face-to-face outreach was initiated during MBMT-2 with a native Spanish speaker with close ties to the community. This kind of targeted recruitment may be necessary for other studies of similar conditions and populations to ensure a study population that is more representative of the general population at risk.

In terms of study limitations, comparisons among the strategies about recruitment success should be interpreted with caution given that the recruitment intensity, time duration, resources and efforts of study staff for each strategy were not systematically measured. Another study limitation is that it was not possible to determine what percentage of women who received information about the study were actually study eligible. For MBMT-1 and 2 recruitment, we used several population-based recruitment approaches to identify potential participants who were relatively difficult to reach. Posters on a bus line or flyers in a church can reach many individuals, but did not allow us to assess how many individuals saw recruitment materials. As such, we were not able determine our denominator- those who encountered the recruitment materials. If a woman called into the 211 referral line, there are call records of them having been referred to the study in call records. In future studies, it would be beneficial to determine what percentage of informed women actually applied to and were successfully enrolled into the study. Another limitation was the missing socio-demographic data among participants recruited during Phase 1. In Phase 1, most demographic information was collected via a questionnaire with participants after they had been mailed the self-collection kit. Demographic information is available only for individuals who were not lost to follow up before that point. This issue was addressed in Phase 2 of the study, as this demographic information was collected earlier in the process, during the initial eligibility screening call.

Both Phases 1 and 2 of the My Body My Test study resulted in relatively high self-collection kit return rates compared to other studies of a similar nature. Study staff implemented five different recruitment strategies to recruit a population of consistently underrepresented individuals. Implementation science researchers should continue to compare different recruitment strategies and to report on their success at recruiting underserved populations to ensure a diverse study population. Effectively recruiting a diverse study population is crucial in the effort to lower cervical cancer incidence and mortality in the populations that carry the highest burden.
